# Supplementary material for: Influence Maximization for Fixed Heterogeneous Thresholds
Source: Sci Rep. 2019 Apr 3;9:5573. doi: 10.1038/s41598-019-41822-w (PMC6447584; doi:10.1038/s41598-019-41822-w)
Supplement: Supplementary file 1 — Supplementary Information: Influence Maximization for Fixed Heterogeneous Thresholds [file 41598_2019_41822_MOESM1_ESM.pdf]

Supplementary Information: Influence Maximization for Fixed Heterogeneous Thresholds

P. D. Karampourniotis[1,2]*, B. K. Szymanski[2,3,4], G. Korniss[1,2]

February 19, 2019

[1] Department of Physics, Applied Physics, and Astronomy, Rensselaer Polytechnic Institute, 110 8th Street, Troy, NY, 12180-3590 USA
[2] Social Cognitive Networks Academic Research Center, Rensselaer Polytechnic Institute, 110 8th Street, Troy, NY, 12180-3590 USA
[3] Department of Computer Science, Rensselaer Polytechnic Institute, 110 8th Street, Troy, NY, 12180-3590 USA
[4] Faculty of Computer Science and Management, Wroclaw University of Science and Technology, Poland

Influence Maximization against average degree

In addition to the network measure of degree assortativity, we also compare here the perfomance of strategies against the average degree $\langle k \rangle$ for ER graphs, see Fig. S1. We examined the minimum required fraction of initiators required to activate S_{goal}=0.2 of nodes (we consider the initiators to be part of the initially activated set). Regardless of the standard deviation used, GPI is the best performing strategy, while BI is the second best strategy, but the latter only marginally better than CI-TM for $\sigma = 0$ (for this S_{goal}=0.2). We should notice that for low σ, p_c is increasing with average degree, while for larger σ we observe the opposite trend. With the clear exception of GPI, for $\sigma = 0$ most strategies are not effective against high degrees, were each additional seed is in fact contributing with marginal spread.

Influence Maximization on empirical networks

We further examined the performance (Fig. S2) of all strategies against empirical (social and collaboration) networks selected from SNAP [1] with details about them listed in Table SS1. Interestingly, here BI strategy is the best for the three of the empirical networks, while still ranking high on the last 'facebook-combined' network. Although the GPI strategy is out-performing other strategies for lower initiator fractions, it is falling behind for higher ones, yet it can always be improved by controlling its parameters, the granularity of initiator set s and the number of simulations v.

*E-mail: karamp.pan@gmail.com

Table S1: Empirical network statistics

network	N	$\langle k \rangle$	k_{max}	ρ	avg clustering coefficient
facebook-combined	4039	43.7	1045	0.5412	0.61
p2p-Gnutella05	8846	7.2	88	0.0159	0.01
CA-HepPh	12008	19.7	491	0.7172	0.61
CA-CondMat	23133	8.1	280	0.2567	0.63

Additional information about probability area plot (Figure 4 of paper)

In Fig. S3, we provide additional information about the are plot (Fig. 4 of the paper).

BI optimal weights as a function of ρ

We further demonstrate (Fig. S4) that the optimal weights shift as the degree assortativity of the network changes. Interestingly, coefficient c is again kept relatively low regardless of assortativity (we observe similar results for the thresholds' standard deviation σ in Fig. 6 of the paper). We observe that as assortativity increases, so does the importance of the degree of each node for influence maximiziation.

References

[1] Stanford Network Analysis Project (SNAP), http://snap.stanford.edu/data (Accessed: 10/14/2018).

Figures & Captions

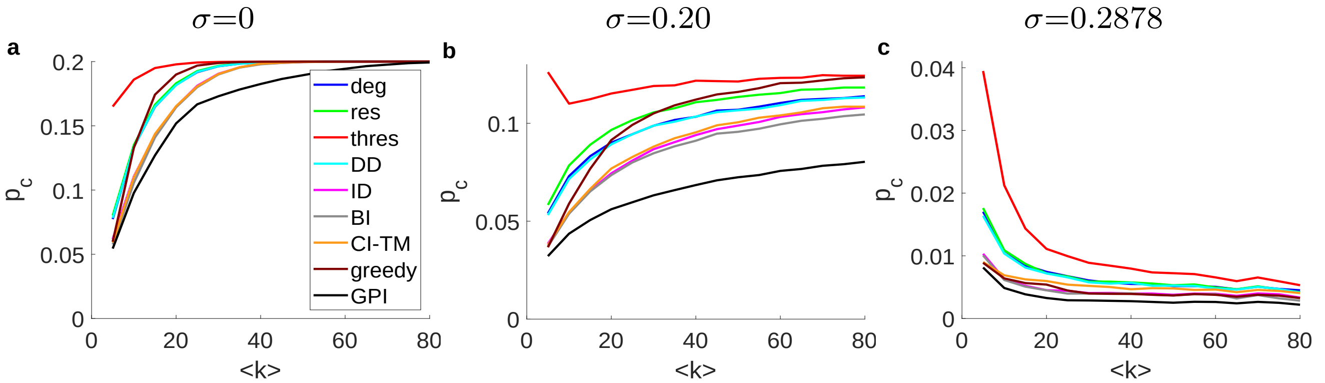

Figure S1: Initiator fraction p_c required to reach spread S_{goal}=0.2 vs. varying average degree $\langle k \rangle$ for ER graphs with N=5,000, with threshold distribution of $\overline{\phi}$=0.5 and (**a**) σ=0, (**b**) σ=0.2, (**c**) σ=0.2887. GPI parameters used here are s=0.001, and v=50,000 (hence v=10∗N). All results are averaged over 30 unique networks and list of thresholds.

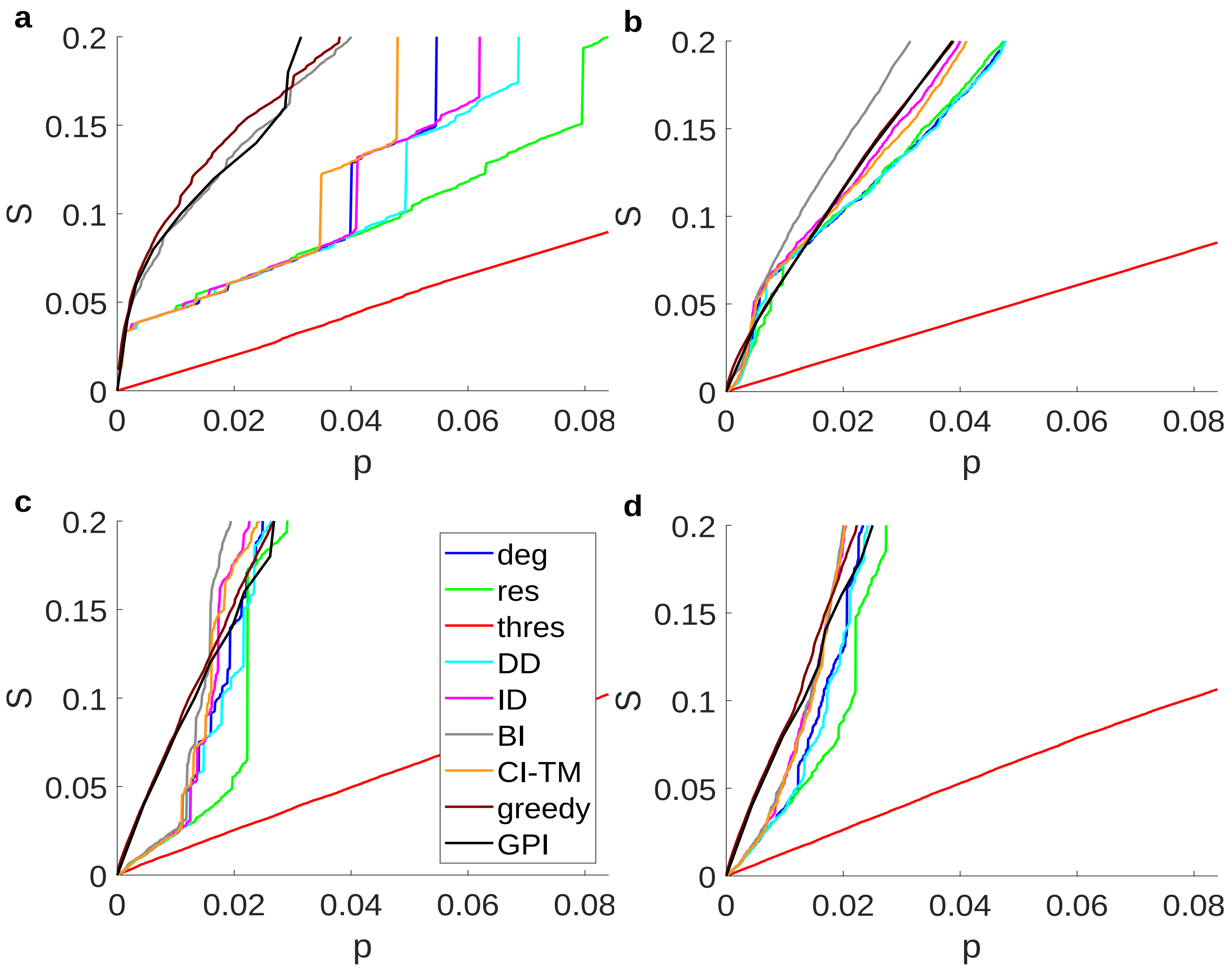

Figure S2: Comparison of cascade performance S_{eq} for empirical networks: (**a**) facebook-combined, (**b**) p2p-Gnutella05, (**c**) CA-HepPh, and (**d**) CA-CondMat, with threshold distribution of $\overline{\phi}$=0.5 and σ=0.2887. GPI parameters used here s=0.001, and v=10 $*$ N.

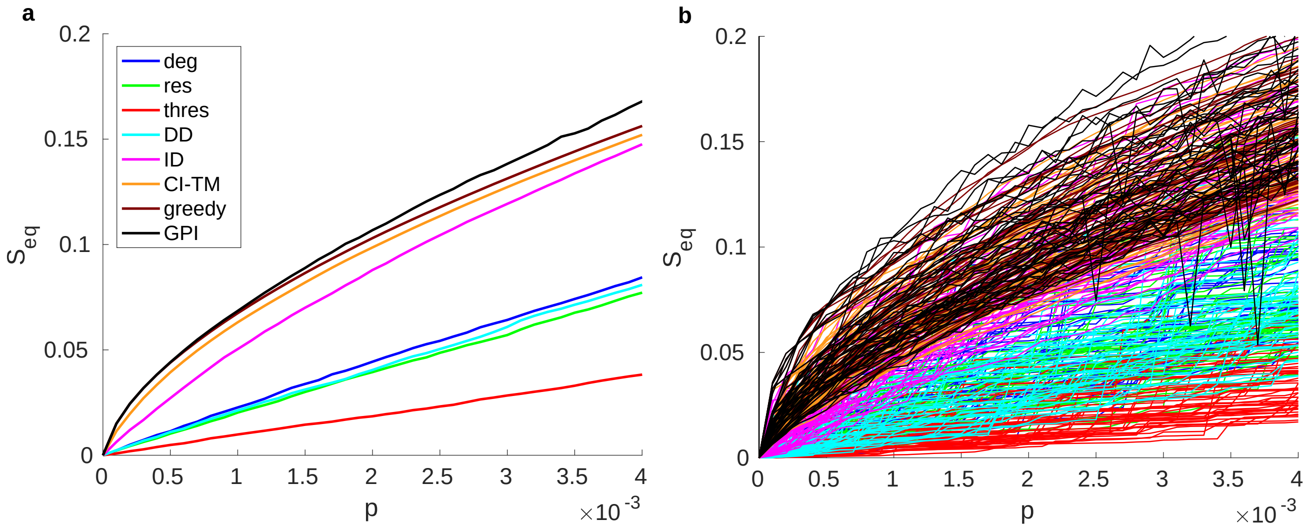

Figure S3: We present the corresponding average (over 240 threshold generations) performance S_{eq} and 50 first runs of each strategy for one ER network (with N=10,000, $\langle k \rangle$=10, and ρ=0.9) for different threshold generations (with $\overline{\phi}$=0.5 and σ=0.2887) in Fig. S3a and Fig. S3b respectively. As is customary, all strategies are compared for the same threshold realizations. These results further support the increasing performance of GPI with increasing initiator fractions.

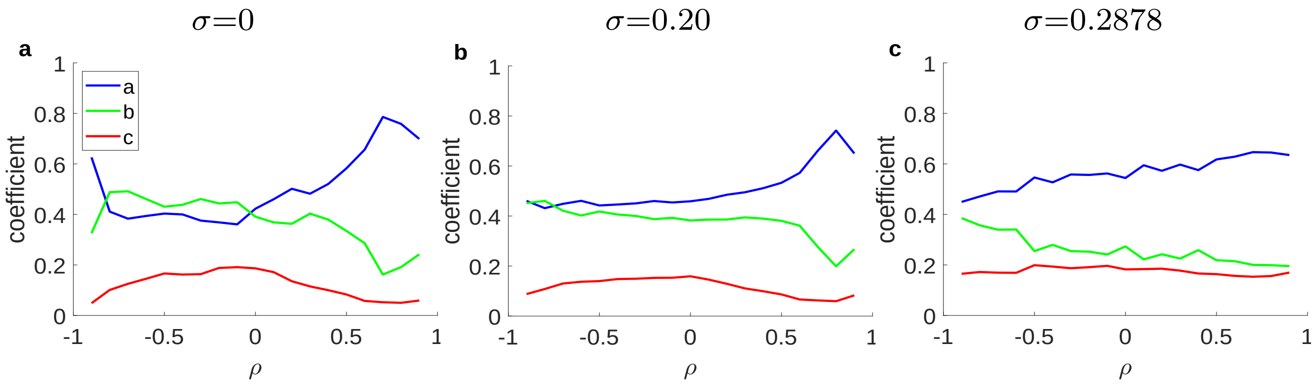

Figure S4: Impact of assortativity on the optimal weights of BI (from Eq. 1), with a+b+c=1) for desired cascade $S_{\rm goal}$=0.5, for graphs with ER degree distribution with N=10,000, $\langle k \rangle$=10, ρ=0, with threshold distribution of $\overline{\phi}$=0.5 and (**a**) σ=0, (**b**) σ=0.2, (**c**) σ=0.2887, averaged for 100 different network realizations each with a different threshold generation. The resolution in the a and b weight space are 0.05.
